# The impact of facial expression and communicative gaze of a humanoid robot on individual Sense of Agency

**DOI:** 10.1038/s41598-023-36864-0

**Published:** 2023-06-21

**Authors:** Maria Lombardi, Cecilia Roselli, Kyveli Kompatsiari, Federico Rospo, Lorenzo Natale, Agnieszka Wykowska

**Affiliations:** 1 Italian Institute of Technology, Via Morego 30, 16163 Genoa, Italy; 2 Italian Institute of Technology, Via Enrico Melen 83, 16152 Genoa, Italy

**Keywords:** Human behaviour, Social behaviour

## Abstract

Sense of Agency (SoA) is the feeling of control over one’s actions and their outcomes. A well-established implicit measure of SoA is the temporal interval estimation paradigm, in which participants estimate the time interval between a voluntary action and its sensory consequence. In the present study, we aimed to investigate whether the valence of action outcome modulated implicit SoA. The valence was manipulated through interaction partner’s (i) positive/negative facial expression, or (ii) type of gaze (gaze contact or averted gaze). The interaction partner was the humanoid robot iCub. In Experiment 1, participants estimated the time interval between the onset of their action (head movement towards the robot), and the robot’s facial expression (happy vs. sad face). Experiment 2 was identical, but the outcome of participants’ action was the type of robot’s gaze (gaze contact vs. averted). In Experiment 3, we assessed—in a within-subject design—the combined effect of robot’s type of facial expression and type of gaze. Results showed that, while the robot’s facial expression did not affect participants’ SoA (Experiment 1), the type of gaze affected SoA in both Experiment 2 and Experiment 3. Overall, our findings showed that the robot’s gaze is a more potent factor than facial expression in modulating participants’ implicit SoA.

## Introduction

Sense of Agency (SoA) is the experience of controlling one’s own actions, and, through them, the course of external events^1^. SoA is a mechanism that is the basis for attributing action consequences to oneself^2^, in such a way that one can have the experience of “making something happen”^1^.

SoA can be quantified using either explicit measures, which require participants to explicitly rate their perceived degree of control, or using implicit measures, which do not involve any explicit inquiry about the agentic experience, and thus they do not rely on conscious reflections vulnerable to subjective beliefs, contextual cues, and differences in personality (^3^; see^4^ for a more complete overview about measures of SoA). One common procedure to measure implicit SoA is time interval estimation^5^, which requires participants to estimate the perceived length of the time interval between two types of events (for example, a voluntary action and its sensory consequence). The result is the *Intentional Binding (IB) effect*, i.e., a subjective compression between the two events, which are perceived as closer in time than they are in reality. As a consequence, the greater the temporal compression, the stronger the experience of SoA (see^6^ for a review).

To date, several studies have elucidated what factors modulate the experience of agency. For example, SoA is now known to be affected by basic sensorimotor processes (e.g.,^7^, as well as high-level cognitive cues, such as intentions and beliefs (e.g.^3^). Another interesting factor potentially modulating SoA is the affective and social dimension, namely, the emotional valence of outcomes produced by individuals’ actions, and the (social) context participants are immersed in.

### Emotional valence of outcomes and SoA: the role of facial expression for the experience of agency

A crucial aspect of SoA is the self-referential processing. More specifically, the agentive experience has been assumed to be mostly guided by self-assessment motives, that is, the internal motivation to gain and use accurate information about the self. However, SoA cannot be reducible only to the contribution of sensorimotor and cognitive cues, but it is also reasonable to hypothesize a tight and reciprocal relationship between SoA and affective processes. This hypothesis is supported by evidence showing that the emotional consequences of one’s actions can influence the experience of agency. For example, Takahata and colleagues (2012) showed that the affective valence of action outcomes has an impact on implicit SoA^8^. With the use of the IB paradigm, the authors asked participants to estimate various tone-reward associations, where auditory stimuli were paired with positive, neutral, or negative monetary outcomes. Results showed that implicit SoA, in the form of the IB effect, was reduced when negative outcomes occurred, i.e., for action outcomes signaling monetary loss^8^. Along a similar line, Yoshie and Haggard^9^ demonstrated that implicit SoA is attenuated for negative emotional sounds, as compared to neutral and positive outcomes.

Facial expressions can also be considered a strong emotional stimulus, since they can convey information related to emotional states. In the context of SoA, previous evidence demonstrated a relationship between the emotional valence conveyed by one’s facial expression and the experience of agency. For example, in a recent study^10^, participants were exposed to video clips showing an actor’s facial expression of varying intensity, to examine whether SoA (and, by extension, personal responsibility for another person’s pain) modulated their empathetic response at both behavioral (i.e., pain intensity estimation from facial expressions, and explicit ratings of unpleasantness), and electrophysiological level (facial electromyography). Results showed that the degree of perceived responsibility, and thus the experience of agency, increased participants’ affective responses (i.e., the rating of unpleasantness); furthermore, the contraction of two facial muscles involved in pain expression also increased with perceived responsibility^10^.

Another study investigated whether the implicit SoA, in the form of sensory attenuation, was affected by outcome valence, namely happy or angry faces^11^. Event-related potential (ERP) results showed that the emotional value of the outcome affected the classical N1 attenuation effect, which is thought to reflect a fast and basic registration of self-agency^12^. Specifically, the authors found a reduced agentive self-awareness for negative (i.e., angry faces) as compared to positive outcomes (i.e., happy faces)^11^. Their results further revealed that the N1 attenuation effect was related to self-serving attributive judgments, but only for negative and not positive outcomes. Specifically, participants with a large negative bias showed less N1 attenuation in response to the negative outcomes, suggesting that SoA is affected primarily by unfavorable outcomes. In other words, this suggests that people are more prone to attribute positive outcomes to themselves, as they contribute to maintain and enhance self-esteem and self-efficacy, which would result in a stronger SoA; on the contrary, negative outcomes would be externally attributed, resulting in a reduced SoA^11^, which is in line with the self-serving bias phenomenon^13^.

However, it is important to mention that there is other evidence showing that the temporal binding, as a proxy measure of participants’ implicit SoA, is not affected by the emotional valence of the action outcome. For instance, Moreton and colleagues^14^ conducted a series of experiments to investigate the degree to which the IB effect is affected by the emotional valence of the outcomes, which were either positive and negative emoticons (Study 1), or positive and negative human facial expressions (Study 2). In both cases, results showed no significant difference in participants’ IB effect related to the type of outcome^14^.

These contrasting results might be potentially explained by the different mechanisms underlying SoA, namely the predictive versus postdictive mechanisms. More specifically, the predictive component refers to the feeling of being in control because it is possible to predict the outcome of a certain action (e.g., I feel in control of my car as I know that, if I press the brake pedal, the car will stop), whereas the postdictive component refers to the possibility of inferring retrospectively the consequences of a given action (e.g., I won the lottery because I retrospectively attributed to myself the ability to choose the right combination of numbers, although it was completely random). In Yoshie and Haggard’s study^9^, the authors found that SoA was reduced for negative outcomes, as compared to neutral and positive outcomes. Notably, in their study the presentation of all three types of outcomes was given in separate blocks, thus making the valence of the outcome entirely predictable, and corroborating previous findings. Furthermore, Yoshie and Haggard replicated the same results in another study^15^, showing that participants’ implicit SoA, in the form of the IB effect, was significantly reduced for predictable negative compared to predictable positive outcomes. In this context, it seems plausible that the predictive component of SoA was primarily affected, although the postdictive component could not be entirely ruled out either. Conversely, when the emotional valence of action outcomes was unpredictable, the IB effect did not significantly differ between the negative and positive outcomes^15^. Similarly, in Takahata and colleagues’ study^8^, the authors found that the IB effect was attenuated when negative outcomes occurred, i.e., outcomes signaling monetary loss. Notably, also in this study the valence of the action outcome was randomized, thus making the association between a tone and the positive, neutral, or negative monetary outcome unpredictable. Therefore, the authors argued that outcome valence might have affected implicit SoA retrospectively^8^.

In another study, Christensen and colleagues^16^ asked participants to perform an IB experiment in which voluntary actions were followed by emotionally positive or negative human vocalizations, or by neutral tones. In this experiment, the authors measured both the predictive component of SoA, driven by the expectation that the outcome will occur, and the postdictive component, triggered by the occurrence of the action outcome. Results showed that, when unexpected, the positive outcome enhanced SoA retrospectively; however, when the outcome valence was blocked (and thus predictable), this postdictive effect was eliminated, and in fact reversed for both positive and negative outcomes, resulting in a reduced SoA^16^.

This said, the current evidence in the literature seems to suggest that the effect of the emotional valence of the outcomes is not always straightforward, and that their impact on SoA can vary depending on mechanisms that are affected by the experimental manipulation.

### Eye contact and SoA: the role of gaze for the experience of agency

Gaze is a powerful social cue playing a fundamental role in the development of social skills in humans^17^. For example, it can be used to engage in joint attention, express and detect others’ emotional and mental states, regulate turn-taking in conversations, or exert social control over others^18^. Therefore, it is not surprising that gaze cues contribute significantly to complex forms of social cognition. Gaze plays also a crucial role in the adoption of visual perspective-taking (e.g.,^19,20^), and theory of mind, as gaze perception seems to activate brain regions, such as the medial prefrontal cortex or the temporoparietal junction (e.g.,^21–23^), that are associated with inferring the mental states of others. Moreover, gaze plays a crucial role also for joint attention, as it informs about readiness to engage in social interactions (e.g.,^24,25^).

One key component of gaze which affects social interactions, and subsequently the corresponding cognitive processes and states, is the direction of the gaze. For example, Senju and Hasegawa^26^ presented on a screen a face with different gaze directions (direct, averted, and closed eyes), followed by a peripheral target. Results showed that participants were slower in detecting the target when the face was directly gazing at the observer (direct gaze), as compared to both averted gaze and closed eyes, suggesting that eye contact delayed attentional disengagement from the face^26^. Along a similar line, Bristow and colleagues^27^ examined whether participants’ behavioral and neural responses to gaze shifts were modulated by the social context of the gaze shift (social: mutual gaze, non-social: averted gaze). The authors found that the eye contact preceding the gaze shift facilitated the detection of the gaze shift, suggesting that participants’ attention was covertly attracted by the social context of the face^27^.

Interestingly, gaze has been also demonstrated to increase self-referential processing (see^28^ for a review). Specifically, its effect on self-awareness has been tested in many experimental contexts. For example, Hietanen and colleagues^29^ investigated whether seeing another person’s mutual versus averted gaze affected participants’ subjective evaluation of self-awareness. Results showed that mutual gaze resulted in an enhanced self-awareness rating by the participants, relative to averted gaze, most likely due to the presence of another person^29^. In a similar vein, Baltazar and colleagues^30^ demonstrated that the perception of a face with a direct gaze (i.e., establishing mutual eye contact), as compared to either a face with an averted gaze or a mere fixation cross, led participants to rate more accurately the intensity of their physiological reactions induced by emotional pictures, as assessed by physiological arousal (skin conductance response)^30^. In this context, a recent study^31^ investigated whether direct gaze, due to its influence on self-referential processing, would affect participants’ implicit SoA, measured by means of the time interval estimation paradigm. The authors asked participants to respond through a button press to a face stimulus, which either established direct gaze with participants (mutual gaze) or not (averted gaze). After each trial, participants’ task was to estimate the time between the keypress and its ensuing effect (mutual vs. averted gaze). Interestingly, results showed a stronger IB effect for mutual as compared to averted gaze, supporting the idea that eye contact modulates implicit sense of agency^31^.

Although many studies showed that perceiving individuals’ direct gaze has strong effects on various attentional and cognitive processes (see^32^ for an extensive review), it is also important to mention that the impact of another individual’s direct versus averted gaze needs to be thoroughly examined, as literature reports results in various, often opposite, directions. For example, in the pivotal study of Ellsworth and Carlsmith^33^, participants were interviewed by an experimenter, who looked directly at the participants’ eyes or their left or right ear. In addition, the verbal content of the interview was manipulated to be either positive or negative. Results showed that, when in the positive context, participants in the direct gaze group evaluated both the interview and the interviewer more positively as compared to those in the averted group. However, results were exactly the opposite in the negative context, namely, the evaluation was more positive in the averted relative to the direct gaze group^33^. In a more recent study^34^, the authors investigated the so-called Temporal Order Judgments (TOJs) of gaze shift behaviors, and evaluated the impact of gaze direction (i.e., direct vs. averted gaze) on TOJs performance measures. Results showed that averted gaze shifts are prioritized over direct ones, as they could potentially signal the presence of behaviorally relevant information in the environment^34^. Interestingly, these results are in line with those of Abubshait and colleagues^35^, which used Transcranial Magnetic Stimulation (TMS) to investigate the social effects of communicative gaze (i.e., direct vs. averted gaze) on attentional orienting. Participants were asked to complete a gaze cueing task with the humanoid robot iCub^36^, which engaged either in mutual or averted gaze before shifting its gaze. Results showed that averted gaze elicited a gaze-cueing effect, whereas the mutual gaze condition did not^35^. Although previous studies showed that mutual gaze facilitates attentional orienting (e.g.,^37–40^), maybe due to its strong, communicative intent^41^, there is other evidence showing that averted gaze stimuli induce strong cueing effects^33–35^. In fact, it can also communicate intent for interaction^42^, and signal turn-taking^43^. Moreover, viewing faces with direct, and even more with averted gaze (as compared to those with eyes closed), activates some of the brain areas that are involved in tasks requiring the attribution of other people’s intentions and beliefs (e.g.,^21,44^). Therefore, it seems that both mutual and averted gaze can be interpreted as communicative gaze, i.e., as a strong social signal being able to communicate intentions to others.

To date, the impact of emotional valence and gaze contact on SoA, both in terms of separate factors and their combined effect, has not yet been extensively studied. Moreover, it has been studied with 2D stimuli presented on the screen. As both these factors have a strong social nature, it is important to ask the question of whether, and to what extent, they affect SoA when the interaction partner exhibiting these signals is physically embodied and present in the physical environment of the participant. According to the “second person neuroscience” perspective^45^, interactive—rather than observational—protocols are the key to understand social cognition. With this in mind, we set out to study the impact of emotional valence and gaze contact on SoA in an interactive protocol with a physically embodied interaction partner. We resorted to using a humanoid robot as a proxy of another human interactor, in order to maintain excellent experimental control, which is difficult to achieve in human–human interaction studies.

Another novelty in our approach, and a move towards a more ecologically valid protocol, was the use of natural actions rather than artificial button presses. To date, the SoA literature has examined arbitrary sensory consequences (e.g., computer-generated sounds) of artificial actions, such as button presses (i.e., the pivotal study of Haggard and colleagues^46^). However, the social effects of one’s actions in the daily environment usually occur as consequences of more natural actions, such as turning one’s head/gaze towards someone else. With this in mind, we focused on SoA in the context of natural actions of a head movement and a more natural/social consequence thereof (emotional valence, gaze contact, or averted gaze).

## Aims

In three experiments, we examined whether, and how, implicit SoA is modulated by the valence of participants’ outcome, in the form of (i) type of facial expression, and (ii) type of gaze of the interaction partner. In all experiments, we asked participants to perform a time interval estimation task^5,31^ with the humanoid robot iCub^36^. As in the classical time interval paradigm^5^, participants were asked to estimate the time interval between a voluntary action and its outcome. However, in all experiments, we adapted the classical paradigm to a more ecological scenario, in which participants were asked to perform natural movements that they are used to perform in their daily life, such as head movements, rather than artificial button presses. Moreover, in our version of the task, the outcomes of participants’ action had a “social” nature, in contrast to the classical paradigm where the results of participants’ button presses are usually artificial beep sounds^46^.

In Experiment 1, the outcome of participants’ action was the robot’s facial expression, which had either a positive (Happy Face condition) or a negative valence (Sad Face condition). In Experiment 2, the outcome of participants’ action was the robot’s gaze, which was either directed towards participants (Mutual Gaze condition) or away (Averted Gaze condition). In Experiment 3, participants observed all the possible combinations between robot’s type of facial expression (happy vs. sad) and type of gaze (mutual vs. averted), to allow for examining the potential effect of the combined factors on participants’ implicit SoA, in a within-subjects design, and for addressing their contribution to the predictive/postdictive mechanisms regulating SoA.

For Experiment 1, if the mechanisms that our paradigm was tapping onto were primarily predictive mechanisms, we would expect to find no differences, in terms of participants’ temporal estimates, between the two types of outcomes (i.e., robot’s happy vs. sad face), as shown by Yoshie and Haggard’s study^15^. On the other hand, if our manipulation evoked more postdictive mechanisms, we would expect to find a stronger SoA (i.e., lower temporal estimates) when the outcome of participants’ action was positive, namely the robot’s happy face, as compared to the (negative sad face), as in Takahata and colleagues’ study^8^. In fact, such retrospective inference would be strongly linked to the self-serving bias^47^, according to which people tend to attribute a greater agency to themselves over positive, compared to negative outcomes (e.g.,^48,49^).

For Experiment 2, if communicative gaze (i.e., mutual vs. averted) is a relevant factor for individuals’ implicit SoA, we would expect to find a significant effect of the robot’s gaze as the outcome of participants’ action. However, we could not formulate specific hypotheses regarding the directionality of the effect, as literature showed that both mutual and averted gaze can be very evocative and either of them can be interpreted as a communicative, social signal modulating various cognitive and attentional processes (see^32^ for an extensive review).

Finally, since the two previous experiments could not clearly disentangle whether our manipulations tapped onto predictive or postdictive mechanisms of SoA, we designed Experiment 3 to address those mechanisms experimentally, and, therefore, to investigate whether the type of gaze and/or the emotional expression affects predictive or postdictive mechanisms involved in SoA.

## Experiment 1

### Materials and methods

#### Participants

27 right-handed participants were recruited to participate in Experiment 1 (Age range 18–45 years old, M_Age_ = 25.7, SD_Age_ = 7.33, 1 left-handed, 1 ambidextrous, 14 males). The sample size was estimated based on previous studies employing a similar procedure (e.g.,^31^), and on a priori power analysis estimating the sample needed to obtain reliable results. Using G*Power v.3.1.9.1^50^ we estimated that a sample size of N = 24 was needed for sufficient power (β = 0.80), to detect a medium effect size (Cohen’s d = 0.5, α (two-tailed) = 0.05). All participants had normal or corrected-to-normal vision and were naïve about the purpose of the study. The study was approved by the local ethical committee (Comitato Etico Regione Liguria), and carried on in accordance with the ethical standards laid down in the 2013 Declaration of Helsinki. Before the experiment, all participants gave written informed consent; at the end of the experimental session, they were all debriefed about the purpose of the study. They all received an honorarium of 15 € for their participation.

#### Apparatus and stimuli

Since our experiment did not require the capability of a full-body robot, we used the version of the humanoid robot iCub^36^ that has the iCub head mounted on a 3D printed body. The robot was placed at the opposite side of the desk, at a distance of 80 cm from the participant, and mounted on a support of 82 cm high to ensure that its eyes were aligned with participants’ eyes. The iCub embeds two PointGrey Dragonfly2 cameras (right and left eye); only one eye-like camera was used as an input sensor to acquire frames with a resolution of 640 × 480 pixels. We used the right-eye camera, but the left camera could be used equivalently. The iCub robot was connected over a local network to a workstation acting as a server and to another client laptop equipped with an external GPU (NVIDIA GeForce GTX 1080). Specifically, the workstation, equipped with a 21’ inches screen (refresh rate: 60 Hz; resolution: 1920 × 1080), was used to launch and control the experiment over the clients, whereas the graphic processor was used to process the RGB frames acquired from the camera in order to implement iCub’s behaviors. Such a screen was positioned laterally on the desk at a viewing distance of 90 cm from participants, thereby allowing them to perform a clear and well-defined head movement from the screen towards the robot. A second screen, placed outside the cabin where the setup was located, was connected to the client laptop, to allow the experimenter monitoring the participant during the completion of the task.

The middleware YARP (Yet Another Robot Platform)^51^ was used to integrate the different system modules (i.e., iCub’s controller, cameras, server, detection system, and code modules). YARP is an open-source framework consisting of a set of libraries and protocols allowing for different applications to communicate with each other in a peer-to-peer manner (https://www.yarp.it/latest/).

Furthermore, a mouse and a keyboard were provided to participants to give their responses. Stimuli presentation and response collection were controlled using OpenSesame v. 3.3.12b1^52^.

#### Experimental procedure

At the beginning of the experimental session, participants were asked to sit in front of the iCub robot. The screen was placed on the left side of participants, approximately at 80 cm from them. A keyboard and a mouse were placed between the participants and the robot, allowing them to give responses throughout the whole duration of the task.

#### Trial sequence

**Figure 1:**
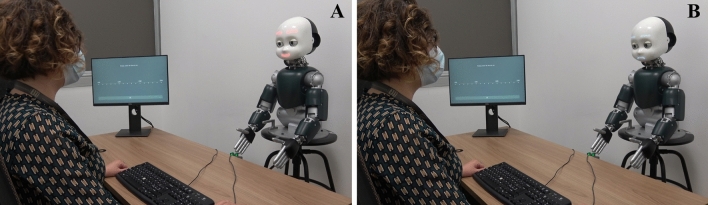
Experiment 1: experimental setup. On the left side (**A**), iCub is shown smiling at the participant as the outcome of her action, i.e., head movement towards the robot (Happy Face condition). On the right side, (**B**), iCub displayed the sad face towards the participant as the outcome of her action (Sad Face condition). Written informed consent for publication of Fig. 1 was obtained.

Each trial started with 700 ms presentation of a white dot in the center of the screen, with a sentence displayed below the dot (“Look at the dot above”), while the iCub robot was in its idle position (i.e., looking slightly down with closed eyes, and showing a neutral facial expression). After the initial time of 700 ms, iCub opened its eyes and moved its head in a frontal position while looking at the participant, always while showing a neutral facial expression (the behaviour lasted 4000 ms: 2000 ms to open the eyes in idle position and 2000 ms to find the human face landmarks and move in frontal position). Participants were invited to look at the dot until a new sentence (“Now you can look at the robot!”) appeared on the screen. Then, participants had to turn their head towards the robot and look it in the eyes, in such a way that the robot could detect if a mutual gaze had been established with them. In the Happy Face condition, once the mutual gaze was established, the robot smiled at participants. Conversely, in the Sad Face condition, the established mutual gaze was followed by the robot displaying a sad face (see Fig. 1). The facial expression of the robot lasted 2000 ms, which was then followed by a blink in both conditions.

**Figure 2:**
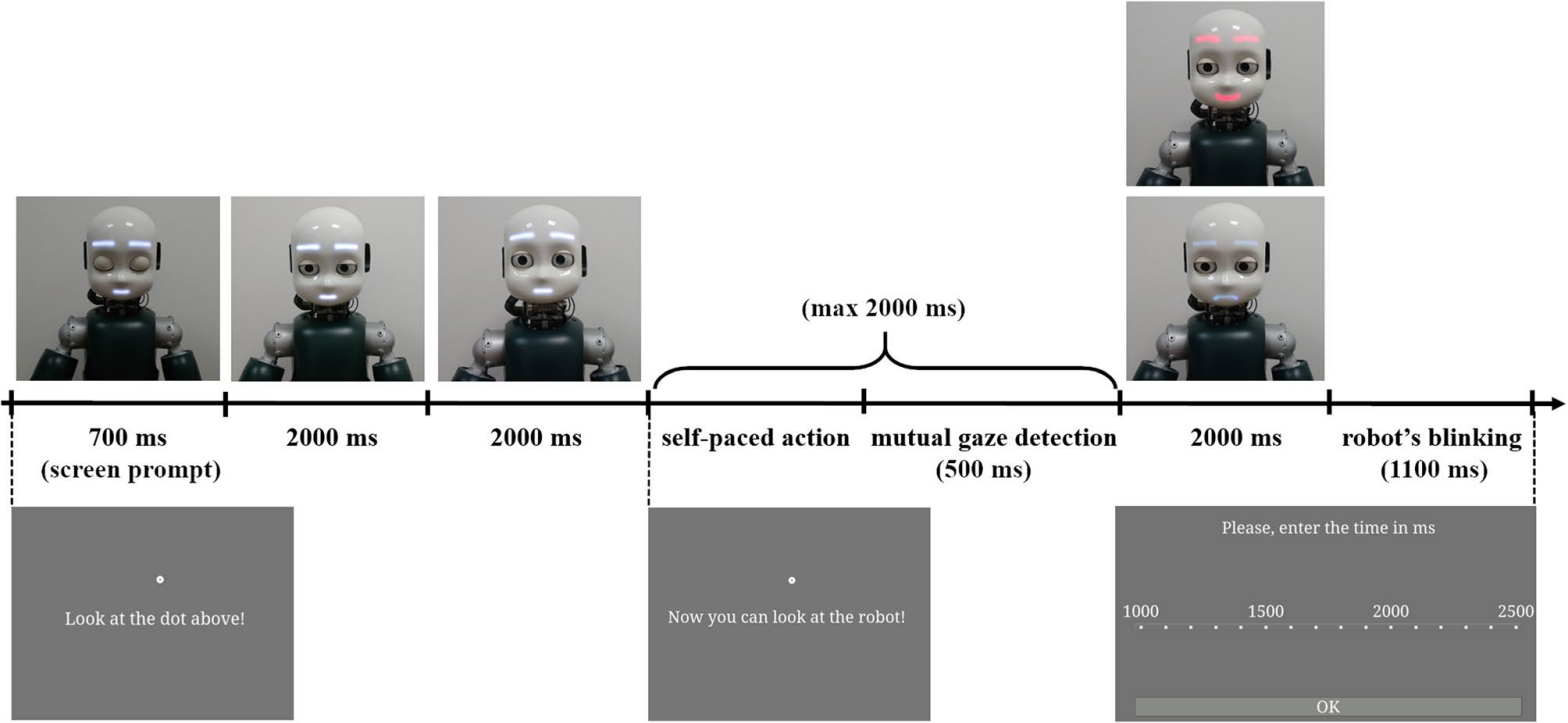
Experiment 1: example of the trial sequence. At the beginning of the trial, the robot was in its idle position. Then, it opened its eyes, raised its head and waited for the action of the participants (i.e., head movement from the screen towards the robot). Once the robot established mutual gaze with participants, it displayed either the happy or the sad face. Note that, if the mutual gaze detection failed (timeout was set to 2 s), the trial still proceeded in such a way that the robot still displayed one of the two expressions, but the trial was marked as invalid. Then, participants gave their responses using the Likert scale.

Then, a Likert-like rating scale appeared on the screen. It ranged between 1000 and 2500 ms, with steps of 100 ms in-between. Participants were asked to rate the time that, according to them, occurred between the moment in which they started to turn the head towards the robot (i.e., onset of the voluntary movement) and the moment in which the robot displayed one of the two expressions (happy/sad face, i.e., the outcome of participants’ action). They were asked to be as accurate as possible, and to not restrict themselves to using only the numbers marked on the rating scale, indicating that they could place the response in-between the 100 ms steps. At the end of each trial, iCub returned to the idle position (see Fig. 2 for an example of a trial).

The task comprised 80 trials for each condition, which were presented randomly throughout the task. A practice session (i.e., 10 trials, 5 for each condition) was administered before the beginning of the task, to let participants familiarize themselves with the instructions and the task. It is important to note that if participants did not turn their head towards the robot within 2 s since the sentence (“Now you can look at the robot!”) was displayed on the screen, the attempt to establish mutual gaze with the robot failed. However, the robot still displayed one of the two facial expressions (happy/sad face), but the trial was marked as invalid and repeated.

#### iCub behavior

The iCub’s behavior consisted of three main modules: (i) the social ability to perceive and recognize when the participant was looking at it (mutual gaze detection); (ii) the motor controller to move the gaze and the head; (iii) the module to implement different facial expressions as reaction to participants’ action.

#### Mutual gaze detection

**Figure 3:**
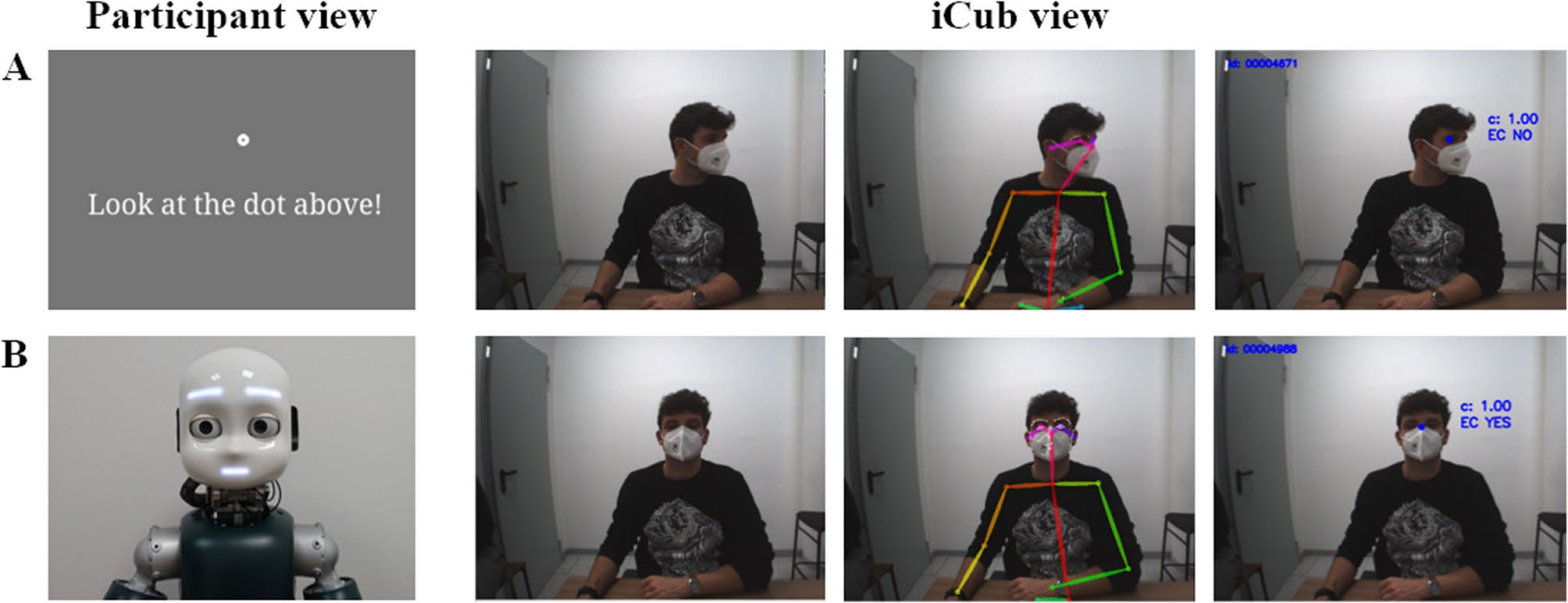
Experiment 1: iCub’s perspective. Participant and iCub views are shown in two different time instants of the experiment: when the participant looked at the screen (**A**) and when she was in mutual gaze with iCub (**B**). In the iCub view on the right side, the RGB frame from the camera, the OpenPose skeleton key-points, and the prediction of the mutual gaze module are shown respectively. Written informed consent for publication of Fig. 3 was obtained.

The participant’s head movement was detected using the mutual gaze detection algorithm proposed in^53^. Such a module connected over the YARP network endows iCub with the social ability to perceive if the participant is in eye contact with it or is still looking at the lateral screen. Briefly, the mutual gaze classifier exploits OpenPose^54^ to extract the feature vector from the participant’s face. OpenPose is a well-known real-time system used for multi-human pose estimation that produces in output the pixel location (x, y) of anatomical key-points for each human in the scene given an RGB frame as input. A subset of 19 face key-points was considered as the feature vector (8 points for each eye, 2 points for the ears, and 1 point for the nose). Then, a binary SVM classifier was trained to recognize events of eye contact/no eye contact with the corresponding confidence level for each prediction (see Fig. 3). According to^53^, the mean accuracy evaluated on 5 different test sets reached a value of 0.91 ± 0.03. During the experiment, a total of three frames were sent in input to the mutual gaze classifier to make its prediction for each frame. The final classifier result was given by a majority rule. That mechanism was implemented in order to avoid flickering in the predictions and be robust to temporary failures. In this setting, the mutual gaze detector produces a prediction each of 500 ms (majority rule on 3 frames at a frame rate of 6 fps).

#### Gaze/head controller

The YARP module iKinGazeController was used, as 6-dof gaze controller (azimuth, elevation and vergence for the eyes and roll, pitch, yaw for the neck), to move iCub’s eyes and neck. Such a module allowed us to independently control eyes and head movement following always the minimum jerk trajectory in order to make it more naturalistic. Specifically, the vergence and the azimuth were set respectively to 3.5 and 0.0 degrees and maintained constant for the whole experiment. To make sure that the head movements of the robot were kept equal across the conditions of interest, we implemented the following three different gaze directions:

- in the resting state the robot looked slightly down and precisely with the elevation component equal to − 15 degrees from its frame of reference in the neck;
- in the eye contact condition the robot looked at the participant’s eyes based on the output given by the face extraction algorithm (publicly available at https://github.com/robotology/human-sensing/tree/master/faceLandmarks). This amounted to elevation of its head to − 5 degrees on average, due to the fixed position of participants on the chair in front of the iCub. In case of detection failure, the robot was programmed to look straight with an elevation of − 5 degrees. This head movement was thus programmed to amount to the difference of 10 degrees upwards from the resting position;
- When the robot looked down (averted gaze) the elevation was set to -40 degrees but blocking the neck pitch to − 25 degrees in order to have the pupils pointing downward. This amount of head movement amounted thus to 10 degrees downwards with respect to resting state position.

Roll and yaw were maintained constant at 0.0 degrees. The pitch component of eye contact/no eye contact condition was equally distanced from the resting state in order to ensure that the distance to reach in both conditions was exactly the same.

#### Facial expressions

Facial expressions were implemented using the iCub faceExpression module allowing us to control the configuration of the eyelids, left and right eyebrows, lip movements, and change the color and the brightness of the face LEDs. Three different emotions were implemented: neutral, happy, and sad face. Specifically, the face LED for the neutral face were white, the LEDs for the sad face were green, while for the happy face they were red. To make the sad face more realistic, the robot’s gaze was set to point at − 24 degrees with the neck pitch blocked at -10 degrees.

### Statistical analyses

#### Data preprocessing

Data of one participant were excluded because the experiment crashed; additionally, data of two other participants were excluded due to a high number of timeouts in one or both conditions. This resulted in a final sample of analysis being equal to N = 24. Subsequently, we excluded all trials marked as “invalid”, i.e., those trials in which the robot failed in establishing mutual gaze with participants before displaying its facial expression (i.e., happy or sad face). Furthermore, we checked for outliers, namely trials deviating more than ± 2.5 SD from participants’ task mean in each block. No outliers were found.

#### Outcome type (robot’s Happy vs. Sad Face) and implicit SoA

To investigate whether participants’ type of outcome, in the form of robot’s facial expression (Happy vs. Sad Face) modulated their implicit SoA, operationalized as temporal estimates given by participants during the task, we compared the mean values of temporal estimates across the two conditions (Happy vs. Sad Face). We first performed a Shapiro–Wilk test, to assess whether data met the assumption of normality. Since the data were not normally distributed (*p* < 0.001), we performed a non-parametric Wilcoxon signed-rank test in JASP 0.14.1.0 (2020). The threshold of significance level was set at *p* < 0.05; rank-biserial coefficient (r_b_) is reported as an index of the effect size; 95% confidence intervals are reported.

### Results

**Figure 4:**
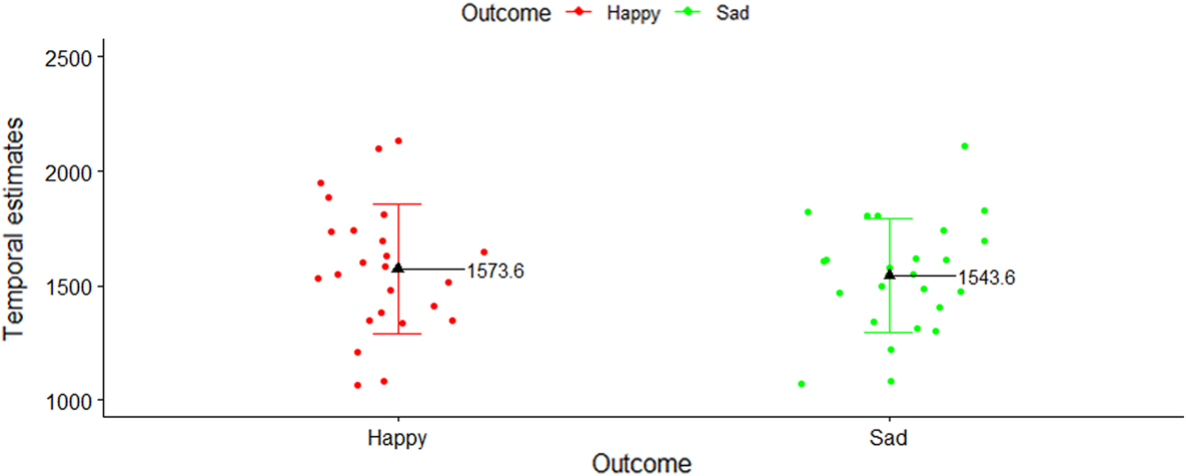
Experiment 1: participants’ mean temporal estimates, plotted as a function of outcome type (Happy vs. Sad Face). Black triangles represent the mean values for each condition, while colored dots represent individual means.

Results showed that the mean values of participants’ temporal estimates did not differ significantly between the two types of action outcome [W = 115, *p* = 0.32, r_b_ = − 0.23, 95% CI (− 0.6; 0.21)] (see Fig. 4).

Additionally, to check whether our results were not biased by the (potentially different) way in which participants used the rating scale to provide their answer (i.e., temporal estimates between 1000 and 2500 ms), we performed the same analysis using *z*- transformed data, to reduce potential inter-subject variability (see^55^ for a similar procedure applied to interval estimates). Results are in line with the main analysis described here and are reported in the Supplementary Materials, paragraph SM.1.1, p. 3).

### Discussion on the role of robot’s facial expression for implicit SoA

The first aim of this study was to investigate whether, and how, the robot’s facial expression, being an emotionally-valenced outcome of participants’ action, modulated their implicit SoA. The implicit SoA was operationalized as participants’ temporal estimates between their action (head movement) and outcome (facial expression of the robot). Participants’ task was to estimate the time interval between the onset of their movement and the onset of robot’s facial expression (happy vs. sad face).

Results showed no significant differences in participants’ temporal estimates between the two types of the action outcome, namely the robot’s happy versus sad face. This suggests that participants’ SoA, at the implicit level, was not modulated by the emotional state conveyed by the robot’s facial expression. This finding can be potentially interpreted in the context of the impact of action outcome valence on predictive versus postdictive mechanisms underlying SoA. Since we did not find the effect of the emotional valence, it might be that our protocol tapped onto predictive mechanisms. Our result would be in line with the study of Yoshie and Haggard^15^, showing that, when the emotional valence of action outcomes was unpredictable, participants’ implicit SoA, in the form of IB effect, did not significantly differ between the positive and the negative outcomes, indicating that the predictive component was primarily affected^15^.

Notably, also in our experiment, the presentation of the two types of robot’s outcomes was randomized (and thus unpredictable for participants). However, as our experimental design did not aim at manipulating the predictive/postdictive mechanisms of SoA, this interpretation needs to be taken with caution. Thus, another possible explanation of why we have not observed the effect might be that the emotional context of the robot’s facial expression was not salient enough to alter participants’ SoA. However, also this interpretation needs to be further addressed in future studies, perhaps with more expressive agents than the iCub robot.

## Experiment 2

### Materials and methods

#### Participants

A new sample of 29 right-handed participants was recruited to participate in Experiment 2 (Age range 18–45 years old, M_Age_ = 23.55, SD_Age_ = 3.43, 3 left-handed, 12 males). The sample size was estimated as in Experiment 1. Similarly to Experiment 1, all participants had normal or corrected-to-normal vision, and were naïve about the purpose of the study. They gave written informed consent prior to the beginning of the experimental session, and the study was conducted under the same ethical protocol as in Experiment 1. They all received an honorarium of 15 € for their participation.

#### Apparatus, stimuli, and procedure

**Figure 5:**
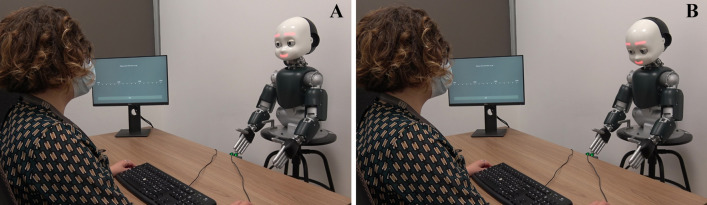
Experiment 2: experimental setup. On the left side (**A**), iCub is shown when establishing mutual gaze with the participant, as the outcome of her action (Mutual Gaze condition). On the right side (**B**), iCub is shown when averting the gaze from the participant, as the outcome of her action (Averted Gaze condition). Written informed consent for publication of Fig. 5 was obtained.

The apparatus, stimuli, and procedure were the same as in Experiment 1, with the only exception that, in Experiment 2, the outcome of participants’ action was the robots’ type of gaze (mutual vs. averted). More specifically, in the *Mutual* Condition, the robot gazed directly at participants, thus establishing mutual gaze, while in the *Averted* Condition, the robot averted the gaze from participants (see Fig. 5). The emotional expression was kept constant across conditions, meaning that the robot always smiled at participants upon their gaze towards its face. Participants’ task was to estimate the time that occurred between the onset of their movement (i.e., head turning towards the robot) and the appearance of the robot’s facial expression (the happy face).

**Figure 6:**
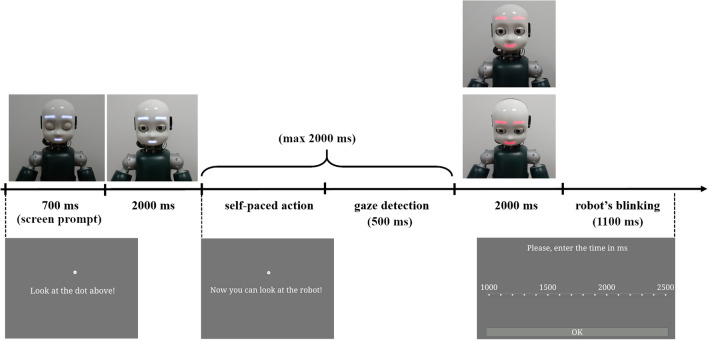
Experiment 2: example of the trial sequence. The robot starts in its idle position (looking slightly down and with closed eyes). Then it opens its eyes without moving its head and waits for the action of the participant. Only when it detects that the participant is looking at it, iCub looks either down (averted gaze) or towards the participants’ face (eye contact) (a timeout of 2 s was set; afterwards, the trial proceeded but marked as invalid). At the end of participants’ head movement, a happy face is displayed for 2000 ms, after that the robot blinks. The screen instructions given to the participants are also shown with the final Likert-like scale.

It is important to point out that the happy face (i.e., the robot smiling) happened immediately after the head movement of the robot (towards or away from participants’ gaze), so it was locked to the end of such a movement (see Fig. 6 for details related to the trial sequence).

The task comprised 80 trials for each condition, which were presented randomly throughout the task. A practice session (i.e., 10 trials, 5 for each condition) was administered before the beginning of the task, to let participants familiarize themselves with the instructions and the task.

It is important to point out that the choice of instructing the participants to judge the time interval between their action (the head movement) and the facial expression of the robot, rather than its gaze (mutual vs. averted) was motivated by the need to have a well-defined time instant for the estimation, and a comparable accuracy in time estimation between the two experiments (facial expression on/off is a discrete event while the eye contact/averted gaze is a continuous process). In addition, asking participants to estimate the time between their action and an event that was orthogonal to the factor of interest (mutual/averted gaze) made the task even more implicit.

#### iCub behavior

The robot behaviors, in terms of (i) mutual gaze detection, (ii) gaze motor controller, and (iii) facial expression, were programmed in the same manner as in Experiment 1.

### Statistical analyses

#### Data preprocessing

Data were preprocessed in the same manner as in Experiment 1. Data of two participants were excluded due to a high number of timeouts in one or both conditions, resulting in a final sample of analysis equal to N = 27. As in Experiment 1, we checked for outliers, and trials deviating more than ± 2.5 SD from participants’ task mean were removed from further analyses (11 trials; M = 1272.72 ms, SD = 806.77 ms).

#### Outcome Type (robot’s Mutual vs. Averted Gaze) and implicit SoA

To investigate whether participants’ outcome of the action, in the form of robot’s type of gaze (Mutual vs. Averted Gaze) modulated their implicit SoA, we compared the mean values of temporal estimates across the two conditions. We first performed a Shapiro–Wilk test, to assess whether data met the assumption of normality. Since data were normally distributed (*p* = 0.29), we performed a paired samples t-test in JASP 0.14.1.0 (2020). The threshold of significance level was set at *p* < 0.05; Cohen’s d is reported as an index of the effect size; 95% confidence intervals are reported.

### Results

**Figure 7:**
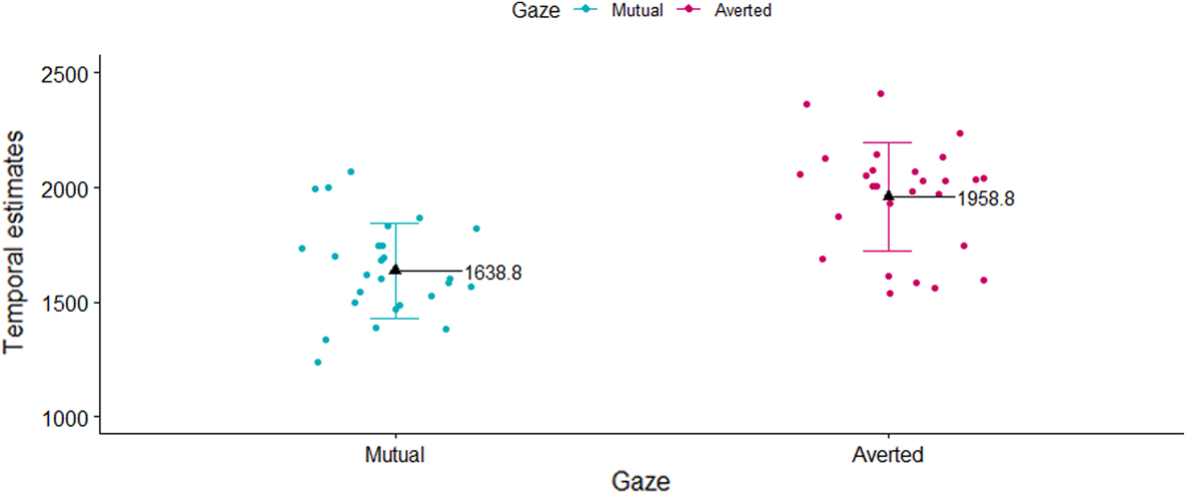
Experiment 2: participants’ mean temporal estimates plotted as a function of outcome type (robot’s mutual vs. averted gaze). Black triangles represent the mean values for each, while colored dots represent individual means.

Results showed that the mean values of participants’ temporal estimates significantly differed as a function of the type of outcome (robot’s mutual vs. averted gaze) [t = 8.69, *p* < 0.001, r_b_ = 1.67, 95% CI (1.07; 2.25)], with lower temporal estimates (i.e., higher implicit SoA) when the outcome of participants’ action was the robot’s mutual gaze, relative to averted gaze (see Fig. 7).

As in Experiment 1, to check whether our results were not biased by the (potentially different) way in which participants used the rating scale to provide their answer (i.e., temporal estimates between 1000 and 2500 ms), we performed the same comparison using *z*- transformed data. Results are in line with the main analysis and are reported in the Supplementary Materials, paragraph SM.1.2, p. 4).

### Discussion on the role of robot’s gaze for implicit SoA

The second aim of the study was to investigate whether, and how, the robots’ type of gaze (mutual or averted), being the outcome of participants’ action, modulated their implicit SoA. To this aim, we employed the same time interval estimation paradigm as in Experiment 1, with the only difference that participants’ outcome was now robot’s type of gaze (mutual vs. averted), rather than emotional expression, which, in Experiment 2 was happy and kept constant across conditions. Participants’ task was to estimate the time interval between the onset of their action, and the robot’s happy facial expression.

Results showed that participants reported lower temporal estimates (i.e., stronger implicit SoA) when the outcome of their action was robot’s mutual gaze, relative to averted gaze. This is an interesting result, showing that gaze type as an action outcome modulates participants’ implicit SoA. Indeed, communicative gaze has been demonstrated to be a powerful social signal modulating various aspects of cognitive processing such as social attention, arousal, and memory^32^. Interestingly, the communicative intent of the gaze has been extensively demonstrated for direct gaze^37–40^, presumably as a consequence of self-referential processing^32^.

This is in line with our results, showing a stronger effect of the mutual gaze on participants’ SoA. However, our findings from this protocol cannot allow us to disentangle whether it is the predictive or the postdictive component of SoA that was affected by the communicative gaze.

Therefore, we designed Experiment 3, to assess whether the type of gaze and/or emotional expression, as outcomes of participants’ actions, affected the predictive of postdictive mechanisms of SoA (or both).

## Experiment 3

### Materials and methods

#### Participants

Forty-two right-handed participants were recruited to take part in Experiment 3 (Age range: 18–45 years old, M_Age_ = 25.6, SD_Age_ = 5.5, 2 left-handed, 1 ambidextrous, 16 males). The sample size was estimated based on a priori power analysis performed with G* Power v.3.1.9.1^50^, to estimate the sample needed to obtain reliable results. Since the present experiment aimed to assess the combined effect of the robot’s type of Emotion and the type of Gaze as outcomes of participants’ action, the experiment was designed as a 2 (Emotion: happy vs. sad face) × 2 (Gaze: mutual vs. averted) within-subject design. Therefore, we estimated that, to perform a Repeated Measures ANOVA, within factors, a sample size of N = 35 was needed for sufficient power (β = 0.80), to detect a small effect size (Cohen’s d = 0.2, α (two-tailed) = 0.05). We finally tested 42 participants, to account for the possible need of excluding participants from further analyses.

Similarly to Experiment 1 and 2, all participants had normal or corrected-to-normal vision, and were naïve about the purpose of the study. At the beginning of the experimental session, they gave written informed consent, and the study was conducted under the same ethical protocol as in the previous experiments. All participants received an honorarium of 15 € for their participation, and they were debriefed about the purpose of the study at the end of the experimental session.

#### Apparatus and stimuli, and procedure

The apparatus and the stimuli were the same as in the previous experiments. Participants were asked to perform the task, which comprised four blocks of 40 trials each (order randomized), for a total amount of 160 trials. Within each block, the two factors (i.e., Type of Emotion vs. Type of Gaze) were combined in such a way that one was constantly presented throughout the entire duration of the block, whereas the presentation of the other one randomly varied during the block. In other words, when the blocked factor was the robot’s facial expression, the robot always displayed the sad or happy face (Block 1), while the gaze was mutual or averted, randomized across trials of that block. On the contrary, when the blocked factor was gaze type, the robot always displayed either mutual or averted gaze throughout the entire block and showed happy or sad face in a randomized manner across trials of a given block (see Table 1 for a summary of the structure of blocks).

**Table 1:** Experiment 3: summary of the structure of each block.

Block	Blocked factor	Randomized factor
1	Sad face	Mutual versus averted gaze
2	Happy face	Mutual versus averted gaze
3	Averted gaze	Happy versus sad face
4	Mutual gaze	Happy versus sad face

Please note that “blocked factor” refers to the factor (i.e., type of Emotion vs. type of Gaze) which was kept constant for the entire block, whereas “randomized factor” refers to the factor whose presentation randomly varied across trials of the block.

#### Trial sequence

**Figure 8:**
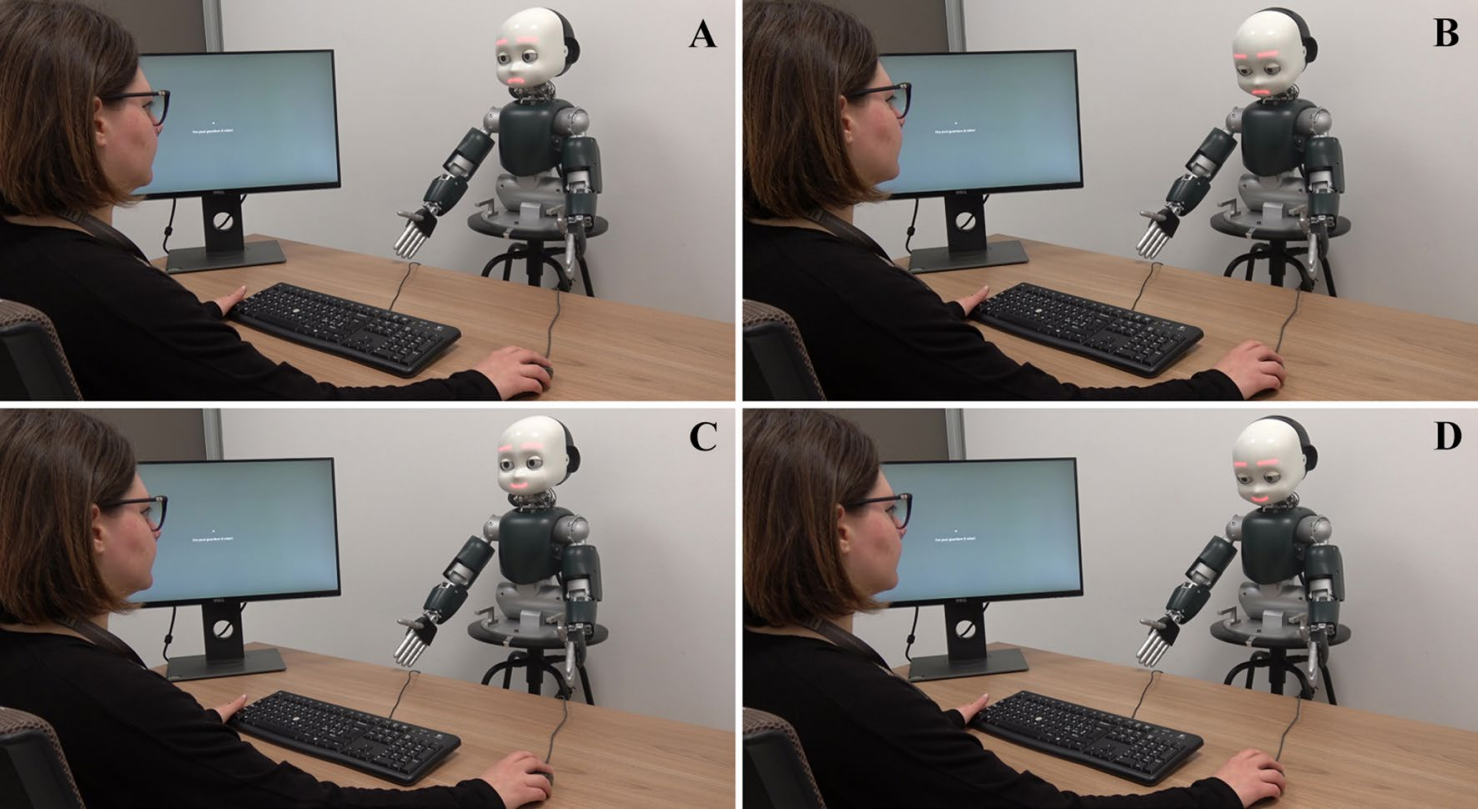
Experiment 3: experimental setup. All possible combinations of type of Emotion (happy vs. sad face) and type of Gaze (mutual vs. averted) are shown. (**A**) sad face + mutual gaze. (**B**) sad face + averted gaze. (**C**) happy face + mutual gaze. (**D**) happy face + averted gaze. Written informed consent for Publication of Fig. 8 was obtained.

Each trial started with a 700 ms presentation of a white dot in the center of the screen with a sentence displayed below the dot (“Look at the dot above”), while the iCub robot was in its idle position (i.e., looking slightly down with closed eyes, and showing a neutral facial expression). After the initial time of 700 ms, iCub opened its eyes always while showing a neutral facial expression (2000 ms). Participants were invited to look at the dot until a new sentence (“Now you can look at the robot!”) appeared on the screen. Then, participants had to turn their head towards the robot and look it in its eyes, in such a way that the robot could detect the participants’ gaze. Afterwards, depending on the block, the robot either moved its head to establish mutual gaze with the participants or averted its gaze by looking down, while displaying either a happy or a sad face. The robot behaviour was timed in order to last constantly 1200 ms (see Fig. 8).

Then, the robot was programmed to say “Go”, which was the signal for participants to judge the timing of, and to turn again their head towards the screen, when a Likert-like rating scale appeared. The scale ranged between 0 and 2500 ms, with steps of 100 ms in between. Using the scale, participants were asked to rate the time that, according to them, occurred between the moment in which they started to turn their head towards the robot (i.e., onset of voluntary movement), and the moment in which the robot said “Go”. They were asked to be as accurate as possible, and to not restrict themselves to using only numbers marked on the rating scale, indicating that they could place the response in-between the 100 ms steps. Then, iCub returned to its idle position.

**Figure 9:**
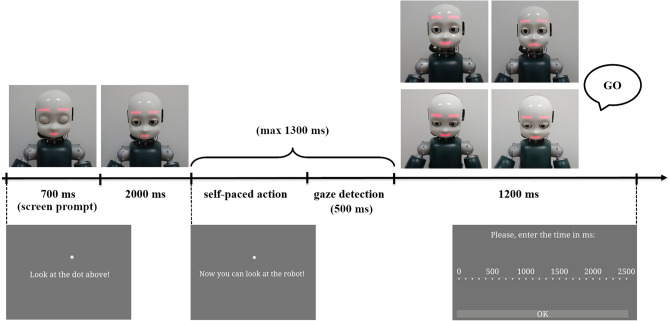
Experiment 3: example of the trial sequence. The robot starts in its idle position (looking slightly down and with closed eyes). Then it opens its eyes without moving its head and waits for the action of the participant. Only when it detects that the participant is looking at it, iCub looks either down (averted gaze) or towards the participants’ face (eye contact) while displaying either a happy or sad face (a timeout of 1.3 s was set; afterwards, the trial proceeded but marked as invalid). At the end of participants’ head movement, iCub says the word “Go”. The screen instructions given to the participants are also shown with the final Likert-like scale.

Notably, both at the end of each trial and at the end of each block, another rating scale appeared on the screen, to ask participants to identify the type of Emotion (happy vs. sad face) and the type of Gaze (mutual vs. averted) displayed by the robot. When asked to evaluate the type of emotions, three options were given (“happy”, “sad”, and “neutral”), with the order of response options on the screen always randomized. The same occurred when participants had to evaluate the type of gaze, with the only difference that the response options were the following: “yes”, “no”, or “I don’t know” (participants were specifically asked whether the robot was looking at them). It was made to check if participants correctly identified the type of emotion versus the type of gaze throughout the completion of the task (Fig. 9).

A practice session (i.e., 8 trials, two for each combination of type of Emotion and type of Gaze) was administered before the beginning of the task, to let participants familiarize themselves with the instructions and the task. It is important to note that, as in the previous experiments, if participants did not turn their head towards the robot within 1.3 s since the sentence (“Now you can look at the robot!”) was displayed on the screen, the attempt to establish the mutual gaze with the robot failed. However, the following events of the trial sequence still occurred, but the trial was marked as invalid and repeated (“timeout”). The timing was carefully tuned reducing the mutual gaze timeout to 1300 ms in order that the time elapsed between the head onset and the robot’s speech can be at maximum 2500 ms (upper bound of the Likert scale). Notably, if participants performed 40 invalid trials, the experiment was automatically interrupted.

#### iCub behavior

The robot behaviors in terms of mutual gaze detection and gaze motor controller were programmed in the same manner as in Experiment 1 and 2. The facial expressions, instead, were changed in order to avoid any potential confound between the emotion itself, the robot’s head position, and other factors like the LEDs’ color and brightness.

#### Facial expressions

Facial expressions were implemented using the iCub faceExpression module allowing us to control the configuration of the eyelids, left and right eyebrows, lip movements and change the color and the brightness of the face LEDs. Compared to the Experiment 1 and 2 where both LEDs color and brightness were changed depending on the robot’s expression, in Experiment 3 the LEDs were fixed to a red color with a medium brightness. Furthermore, we removed the changing in robot’s head direction during the sad face (implemented in Experiment 1 to make it more realistic). Briefly, the robot’s expression was modulated only changing the lip movements in a happy or sad face, maintaining head orientation constant.

### Statistical analyses

#### Temporal estimates

##### Data preprocessing

Data from seven participants were excluded due to a high number of invalid trials during the experiment (i.e., 40 timeouts). This resulted in a final sample being equal to N = 35. Subsequently, trials where participants did not correctly identify the emotion and/or gaze displayed by the robot were excluded from further analyses (7.5% of valid trials; M = 961.19 ms, SD = 526.07 ms). Finally, as in the previous experiments, we checked for outliers, and trials deviating more than ± 2.5 SD from participants’ task mean in each block were excluded from further analyses (22 trials; M = 2500 ms, SD = 0 ms).

To determine whether the type of emotional expression (happy vs. sad face) and/or the type of gaze (mutual vs. averted) affected predictive of postdictive mechanisms involved in SoA, we performed two separate 2 (Emotion: happy, sad) X 2 (Gaze: mutual, averted) Repeated Measures ANOVAs in JASP 0.14.1.0 (2020), for blocked and randomized conditions separately. Participants’ temporal estimates were the dependent variable, and the type of emotion and gaze were the within-subjects factors. The threshold for level of significance was set at *p* < 0.05; η^2^ is reported as an index of the effect size.

##### Separate comparisons for each block

Furthermore, we decided to compare the mean values of participants’ temporal estimates separately for each block, to investigate whether there were any differences, in terms of participants’ temporal estimates (and thus implicit SoA), across all possible combinations between Blocked and Random factors. For each comparison, we first performed a Shapiro–Wilk test, to assess whether data met the assumption of normality. When data were not normally distributed (*p* <  = 0.05), we performed a non-parametric Wilcoxon signed rank test in JASP 0.14.1.0 (2020). The threshold of significance level was set at 0.05; rank-biserial coefficient (r_b_) is reported as an index of the effect size; 95% confidence intervals are reported. Conversely, when data met the assumption of normality (*p* > 0.05), we performed a paired-samples t-test in JASP 0.14.1.0 (2020). The threshold of significance level was set at.05; Cohen’s d is reported as an index of the effect size; 95% confidence intervals are reported.

#### Rating responses

As previously mentioned, at the end of each trial participants were asked to use a rating scale to identify the type of Emotion (happy vs. sad face) and the type of Gaze (mutual vs. averted) displayed by the robot throughout the task. To assess participants’ rate of correct responses across different conditions (robot type of Emotion (happy vs. sad) and type of Gaze (mutual vs. averted)), we computed the rate of participants’ correct responses, and compared them with two separate chi-square tests in JASP 0.14.1.0 (2020), for block and randomized conditions separately. In more detail, the first chi-square test was performed considering the type of Emotion as the blocked factor, whereas the type of gaze randomly varied across trials (and participants were asked, at the end of each trial, to identify the kind of gaze displayed by the robot). Conversely, in the second chi-square test, the type of Gaze was considered as the blocked factor, whereas the type of emotion randomly varied across trials (and participants were asked, at the end of each trial, to identify the type of emotion displayed by the robot). In the subsequent section, results are reported accordingly.

### Results

#### Temporal estimates

##### Emotion as the blocked factor and gaze as the randomized factor

**Figure 10:**
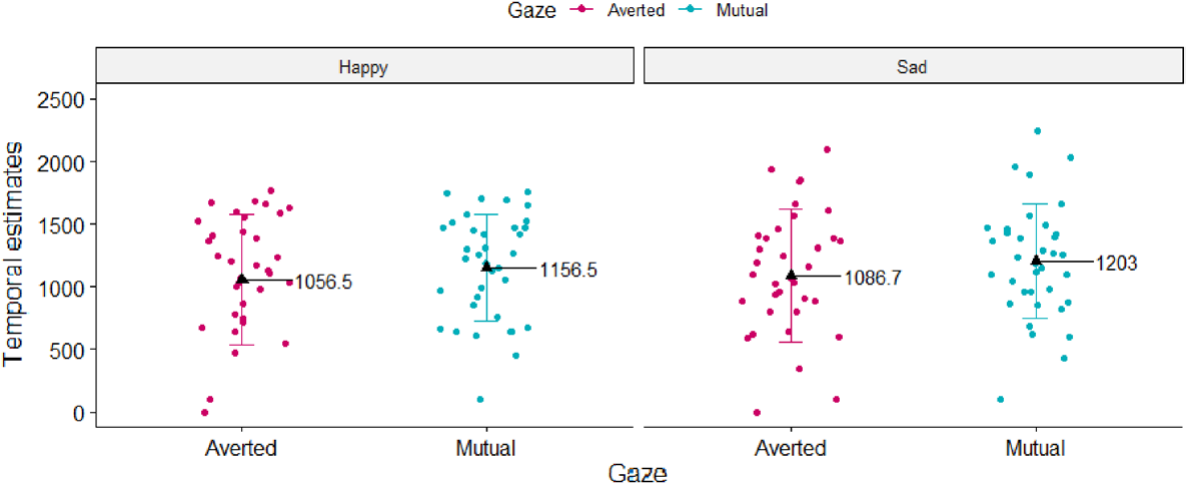
Experiment 3: participants’ mean temporal estimates plotted as a function of robot’s type of Gaze (mutual vs. averted gaze), separately for each type of emotion (happy vs. sad face). Black triangles represent the mean values for each, while colored dots represent individual means.

Results showed that the main effect of Gaze was significant [F_(1, 34)_ = 7.25, *p* = 0.01, η^2^ = 0.08], with lower temporal estimates (i.e., higher implicit SoA) when the outcome of participants’ actions was the averted, relative to the mutual gaze (M_Averted_ = 1072 ms, SE_Averted_ = 78.33; M_Mutual_ = 1180 ms, SE _Mutual_ = 78.33). No other main effects or interactions resulted to be significant (all ps > 0.4) (see Fig. 10).

##### Gaze as the blocked factor and emotion as the randomized factor

Results did not show any significant main effect or interaction (all ps > 0.08).

As in Experiment 1 and 2, to check whether our results were not biased by the (potentially different) way in which participants used the rating scale to provide their answer (i.e., temporal estimates between 1000 and 2500 ms), we performed the same 2X2 Repeated Measures ANOVA as before, but using z-transformed data. Results are in line with the main analyses, and are reported in the Supplementary Materials (paragraph SM.1.3, p. 5).

##### Separate comparisons across blocks

Results showed a significant difference only in Block 1, namely, when the robot constantly displayed a sad face, while mutual versus averted gaze were presented randomly [W = 95.5, *p* = 0.005, r_b_ = -0.59, 95% CI (− 0.8; − 0.26)]. Specifically, participants reported lower temporal estimates (i.e., higher implicit SoA) when the robot’s gaze was averted, compared to mutual (M_Averted_ = 1153 ms, SD_Averted_ = 466.5 ms; M_Mutual_ = 1203 ms, SD_Mutual_ = 456.4 ms). All the other comparisons did not show any significant result (all ps > 0.11).

#### Rating responses

##### Emotion as the blocked factor and gaze as the randomized factor

When the robot type of emotion (happy vs. sad face) was blocked, and the type of gaze (mutual vs. averted) was randomized across trials, results showed no significant differences in participants’ percentage of correct responses (χ^2^ = 1.53, *p* = 0.21; see Table 2 for a summary).

**Table 2:** Percentage frequency of participants’ correct responses (with count of observations within parentheses), reported separately by type of Emotion (blocked factor: happy vs. sad), and by type of Gaze (random factor: mutual vs. averted).

Blocked Factor	Random Factor	Rate of correct responses
Happy	Mutual	96.5% (676)
	Averted	99.2% (695)
Sad	Mutual	93.4% (654)
	Averted	90.7% (635)

##### Gaze as the blocked factor and emotion as the randomized factor

Also in the case when the robot type of gaze (mutual vs. averted) was blocked, and the type of emotion (happy vs. sad) was randomized across trials, results showed no significant differences in participants’ percentage of correct responses (χ^2^ = 0.96, *p* = 0.33; see Table 3 for a summary).

**Table 3:** Percentage frequency of participants’ correct responses (with count of observations within parentheses), reported separately by type of Gaze (blocked factor: mutual vs. averted), and by type of Emotion (random factor: happy vs. sad).

Blocked factor	Random factor	Rate of correct responses
Mutual	Happy	96.8% (678)
	Sad	96.3% (676)
Averted	Happy	83.6% (599)
	Sad	92.1% (645)

### Discussion on the combined effect of robot’s type of emotion and type of gaze for implicit SoA

The aim of Experiment 3 was to investigate whether, and how, the combined effect of robot’s type of emotion (happy vs. sad face) and type of gaze (mutual vs. averted), being the outcomes of participants’ actions, modulated implicit SoA, and whether they affected the predictive or postdictive mechanisms underlying SoA. To this aim, we employed the same interval estimation paradigm as in Experiment 1 and 2, with the only difference that, in Experiment 3, participants were exposed to all the combinations of the factors of interest (mutual vs. averted gaze and happy vs. sad face). Furthermore, in contrast to previous experiments, participants’ task was to estimate the time interval between the onset of their action, and an event that was neutral with respect to the factor of interest, namely the sound of “Go” uttered by the robot. This was done to avoid focusing participants’ attention in the task on one of the factors of interest (gaze type or emotion type). To examine whether our factors of interest impact predictive or postdictive mechanisms underlying SoA, we designed Experiment 3 in such a way that each factor could be blocked or randomized across trials. In some blocks, the type of gaze was blocked while the robot’s emotional expression could vary across trials, while in the other block it was reversed. When a factor was blocked, this outcome could be predicted by participants, and thus, if it showed an effect on temporal estimates, it would mean that it tapped onto predictive processes. Conversely, when it would be randomized and would affect temporal judgments, it would indicate that the factor influenced the postdictive component of SoA.

Notably, at the end of each trial/block, participants were asked to identify, by means of a rating scale, the type of emotion (happy vs. sad face) and the type of gaze (mutual vs. averted) of the robot, in order to check whether participants correctly identified the type of emotion versus the type of gaze throughout the completion of the task. First and foremost, results showed that participants correctly identified the type of emotion versus type of gaze of the robot in the majority of the trials, with no significant differences across all possible combinations of blocked and random factors. Moreover, overall participants reported lower temporal estimates, i.e., they experienced stronger implicit SoA, when the outcome of their action was the robot’s averted gaze, relative to mutual gaze, but only when the gaze type was randomized across trials. The fact that averted gaze elicited lower temporal estimates relative to mutual gaze is in contrast with results of Experiment 2, which were in the opposite direction- namely, participants experienced stronger implicit SoA when the outcome of their action was mutual, relative to averted gaze.

Interestingly, the experimental conditions of Experiment 2 were identical to the ones of Block 2 of Experiment 3, namely, the robot displayed the happy face throughout the entire block, while the type of gaze (mutual vs. averted) randomly varied across trials. Therefore, in Experiment 3 we performed also separate analyses for each blocks, to see whether in Block 2 we replicated the same results as in Experiment 2- i.e., lower temporal estimates (and thus higher implicit SoA) for mutual compared to averted gaze. Results of Block 2 did not reveal any significant difference, in terms of participants’ temporal estimates, between mutual and averted gaze when the robot constantly displayed the happy face.

Instead, we found a significant difference in Block 1, when the robot constantly displayed a sad face while the type of gaze (mutual vs. averted) randomly varied across trials. Specifically, participants experienced higher implicit SoA (i.e., lower temporal estimates) when the outcome of their action was the robot averted gaze, as compared to mutual gaze.

In the context of these analyses, there are two aspects to be considered. First, results of Block 2 (Experiment 3) did not replicate the results from Experiment 2. It might be potentially explained by the fact that the experimental design was slightly different across the two experiments. Specifically, in Experiment 2 the critical event to judge was the robot happy face, namely, a visual positive event. In contrast, in Experiment 3 the critical event to judge was the sound of “Go” uttered by the robot, namely, a neutral auditory event. Moreover, in Experiment 2 participants were asked to judge the temporal interval between their movement and the onset of its outcome (i.e., the robot happy face). Conversely, in Experiment 3 participants judged the temporal interval between their head movement and the offset of the emotional event (note that the “Go” signal occurred at the end of the trial sequence, i.e., when the robot facial expression has been already displayed). It might be of interest for future research to examine the contribution of all these specific factors to the effects of social signals on Sense of Agency.

The second aspect to be considered is that, when analyzing the four blocks separately, we found that, in Block 1 only (i.e., when the robot displayed a sad face throughout the entire block), participants reported lower temporal estimates when the outcome of their action was the robot averted, as compared to mutual gaze. These results mirrored the ones of our main analyses, suggesting that the averted gaze had a stronger effect on participants’ implicit SoA than the direct gaze, when the event to be judged was an auditory “Go” signal. This might be due to the interpretation of such a combined social signal, namely sad face accompanied by the verbal utterance “Go”. Perhaps such social signal is received as more salient than other combinations of the social cues exhibited by the robot.

Overall, these additional analyses did not fully explain the contrasting results between Experiment 2 and 3. However, the opposing results are not entirely surprising, as previous evidence showed that mutual/averted gaze might affect cognitive processes in a contrasting manner, namely, in some experimental setups mutual gaze has a stronger impact^37–40^ while in others, it is the averted gaze that has a stronger influence on participants^33–35^. Perhaps it is the strength/salience of the signal, rather than the meaning of the signal, playing a role in implicit SoA. For a more detailed discussion, see the General Discussion section below.

Regarding the fact that only when the gaze type was randomized across trials, it did affect temporal judgments, it speaks in favor of the idea that the impact of the gaze on SoA has a rather postdictive character, suggesting that postdictive, rather than predictive mechanisms underlying SoA are affected by the gaze type.

Finally, the lack of observed effect of robot’s emotion (happy vs. sad face) suggests that the type of facial expression displayed by the robot did not affect participants’ implicit SoA. It is in line with results of Experiment 1, where no significant differences in participants’ temporal estimates emerged between the two types of outcomes, namely robot’s happy versus sad face. Furthermore, no combined effect of robot’s type of emotion versus type of gaze emerged, due to a lack of significance for the Emotion * Gaze interaction.

Overall, these findings suggest that gaze, relative to emotions conveyed by facial expression, is a more potent factor in modulating participants’ individual SoA, more likely affecting the postdictive, rather than predictive mechanisms underlying SoA. The directionality of the gaze effect was different across experiments: Experiment 2 showed that participants experience a stronger SoA when the outcome of their action was the robot’s mutual gaze, whereas Experiment 3 showed participants’ stronger SoA for robot’s averted gaze. This is an interesting result, suggesting that the effect of gaze direction on SoA is related more to the strength of the signal than to its valence. This is quite plausible, as it might be that the more one is affected by the social signal they “caused” in others (independent on how this signal is interpreted), the more they attribute this signal to their own actions.

## General Discussion

The present study aimed at investigating whether implicit SoA can be modulated by the social valence of action outcomes, in the form of interaction partners’ (i) type of facial expression (Experiment 1), (ii) type of gaze (Experiment 2), (iii) the combination of the two (Experiment 3). Implicit SoA was measured by means of the time interval estimation paradigm^5,31^, whereas the social interaction partner was the humanoid robot iCub^36^.

In three experiments, we asked participants to estimate the time interval between the onset of their natural action (i.e., their head turning towards the robot), and the subsequent outcome, which was manipulated as robot’s happy versus sad facial expression (Experiment 1), mutual versus averted gaze (Experiment 2), or a combination of the two (Experiment 3).

Results of Experiment 1 showed that, when the outcome of participants’ action was the robot’s emotion, conveyed by its facial expression, no significant differences, in terms of participants’ temporal estimates, emerged between the two types of outcomes (happy vs. sad face). However, in Experiment 2, a significant difference was observed as a function of the type of robot’s gaze (mutual vs. averted gaze). Specifically, when the outcome of participants’ action was the robot’s mutual gaze, they reported lower temporal estimates (indicating stronger SoA) as compared to when it was the robot’s averted gaze. Interestingly, when examining the combined effect of the robot’s type of emotion and type of gaze (Experiment 3), results did not show any significant interaction between the two, as indicated by the lack of significance of the two-way Emotion * Gaze interaction. However, it confirmed the main effect of gaze on participants’ SoA, although in the opposite direction compared to Experiment 2: indeed, in Experiment 3 participants’ reported lower temporal estimates (i.e., stronger SoA) when the outcome of their action was the robot’s averted, compared to the mutual gaze. Since the experimental conditions of Experiment 2 and Block 2 of Experiment 3 were similar (i.e., the robot constantly displayed a happy face as the outcome of participants’ action, while the mutual vs. averted gaze randomly varied across trials), in Experiment 3 we also performed separate analyses for each block, in order to see whether results of Experiment 2 were replicated considering only Block 2 of Experiment 3. However, results of Block 2 showed no significant difference, in terms of participants’ temporal estimates, between mutual and averted gaze, and thus results of Experiment 2 were not replicated. However, a significant difference emerged in Block 1, namely, when the robot constantly displayed a sad face while the type of gaze randomly varied across trials. More specifically, we found that participants reported lower temporal estimates (i.e., they experienced a stronger implicit SoA) when the robot averted its gaze, as compared to direct gaze.

One potential explanation might be that the robot facial expression per se is not a factor potent enough to modulate individuals’ implicit SoA. It seems to be confirmed by results of both Experiment 1 and Experiment 3, as no significant main effect of the robot type of emotion (happy vs. sad face) emerged. However, when the facial expression is emotionally congruent with a more potent factor (i.e., communicative gaze), it might act as a reinforcement. In consequence, when the robot averts its gaze for participants (a negative event), and this is accompanied by a sad face (still a negative event), the result is a stronger effect on participants’ implicit SoA, as indexed by lower temporal estimates. If it is the case, in principle we should observe a similar effect also for positive events, namely, mutual gaze (which can signal the readiness and openness to an interaction;^41^) accompanied by a happy face, which we did not find in Experiment 3. However, this is not entirely surprising, as demonstrated by previous findings showing that, at least under certain conditions, negative outcomes have a stronger effect on implicit SoA as compared to positive outcomes^9,15,56^. In either case, this is just a speculative explanation, which needs to be taken with caution and further addressed in future studies.

Another potential explanation of these results might be in the context of the predictive versus postdictive mechanisms underlying SoA.

Regarding Experiment 1, it might be that our paradigm tapped onto predictive mechanisms. Indeed, our results are in line with previous evidence^15^ showing that, when the presentation of emotionally-valenced outcomes was randomized (and thus unpredictable), participants’ implicit SoA, in the form of IB effect, did not significantly differ between positive and negative outcomes. Notably, also in our experiment, the two types of outcomes (i.e., robot’s happy vs. sad face) were unpredictable for participants, as their presentation was randomized across trials. Therefore, it is plausible that the emotional component of the robot’s emotions, conveyed by its facial expressions, did not affect the postdictive component of SoA^9^—namely, the retrospective inference about the outcome of a given action. However, it is important to mention that Experiment 1 was not specifically designed to investigate the role of the predictive/postdictive mechanisms underlying SoA.

Regarding Experiment 2, results showed that the gaze type as the action outcome modulated participants’ implicit SoA, in such a way that participants reported lower temporal estimates (i.e., stronger implicit SoA) when the outcome of their action was robot’s mutual gaze, relative to averted gaze. Our finding is in line with previous evidence demonstrating that communicative gaze, as a powerful social signal, modulates various cognitive and attentional processes, both in form of mutual^37–40^ and averted gaze^33–35^. However, the design of Experiment 2 did not allow us to draw any conclusion about whether the communicative gaze affected predictive or postdictive components underlying SoA.

Therefore, Experiment 3 was designed not only to investigate the combined effect of robot’s type of emotion and type of gaze as the outcomes of participants’ action, but also to determine whether the gaze and/or emotion affects the predictive/postdictive component of SoA. To this aim, Experiment 3 was designed such that each factor could be blocked or randomized across trials. Results showed that participants experienced a stronger implicit SoA, as indexed by lower temporal estimates, when the outcome of their action was the robot averted, compared to mutual gaze. Notably, this was observed only when the gaze type was randomized across trials, and not when it was the blocked factor.

This result is interesting for two reasons. The first interesting aspect is that the direction of the gaze effect of participants’ implicit SoA goes in the opposite direction compared to Experiment 2, where it was the robot’s mutual gaze eliciting a stronger implicit SoA. These findings, apparently at odds with each other, might be potentially explained with reference to the strength/salience of the signal, rather than the meaning of the signal, as a factor playing a role in implicit SoA. The reason why we believe that the strength of the signal might be at stake is that the “Go” utterance in combination with averted gaze (downwards) seemed to have been quite an evocative signal. It might have been a stronger social signal than the “Go” utterance coupled with direct gaze. On the other hand, in Experiment 2, when there was no additional signal in the form of the verbal “Go”, mutual gaze might have been more evocative than averted gaze. Hence, the seemingly “reverse” pattern of results. This speculative interpretation, however, needs to be taken with caution and addressed in future research.

Overall, the effect of averted gaze on SoA is not so surprising, given the “averted-gaze advantage” demonstrated by Riechelmann and colleagues^43^. In three experiments, the authors systematically examined participants’ discrimination performance for gaze direction, namely, they were asked to indicate as quickly and accurately as possible the gaze direction of the presented stimulus via keypress (mutual vs. averted). Their results showed that averted gaze was generally responded to faster and with higher accuracy than direct gaze. This effect was robust and consistent across different (and controlled) experimental conditions, and variations in task demands^43^.

Notably, these findings are in line with previous evidence reporting more averted responses in a gaze-categorization task, compared to direct gaze^57^, and with more recent evidence showing a similar averted-gaze advantage in a gaze-discrimination task^58^. Similarly to our interpretation here, one explanation of the effect was the increased saliency attributed to the averted compared to the mutual gaze, whose saliency has been empirically demonstrated by previous studies (e.g.,^59–63^). This said, it might be that, under certain conditions, also averted gaze can be prioritized over mutual gaze as a powerful social signal being able to affect individuals’ implicit SoA. Concerning this, it might be that the effect of the gaze direction can modulate the observer’s affective responses in different ways according to several factors- for example, the context. To make a practical example, when speaking in front of an audience, a direct gaze usually signals that the listeners are paying attention, with which the speaker might feel more confident and relaxed. Conversely, the audience directing their gaze somewhere else from the speaker might indicate that they are not sufficiently engaged, and thus make the speaker feel anxious and insecure. On the other hand, when in a dark street at night, a person directly looking might evoke negative feelings such as unease and fear, whereas one might feel more relaxed when the same person averts their gaze. This is just a trivial example, but illustrating that the meaning of another person’s gaze can be influenced by several contextual factors. Notably, it is confirmed by evidence in literature, indicating that individuals’ affective reactions elicited by another agent’s direct versus averted gaze appear to result in different, and sometimes opposite, findings (see^32^ for an extensive review). In the present study, the interpretation of the direction of the gaze might have varied, in one experiment resulting in a stronger SoA for robot’s mutual gaze (Experiment 2), and in another for robot’s averted gaze (Experiment 3).

The second interesting aspect of results from Experiment 3 is linked to the predictive/postdictive mechanisms underlying SoA.

The fact that averted gaze elicited lower temporal estimates (namely, stronger implicit SoA) relative to mutual gaze, and only when the gaze type was randomized across trials, suggests that the impact of gaze on SoA has a rather postdictive character, meaning that it affects postdictive, rather than predictive mechanisms of SoA. This is also in line with Experiment 2 where the gaze manipulation was also randomized across trials. It has already been demonstrated that, in situations of low statistical predictability, SoA appears to be driven by postdictive mechanisms based on positive affective surprise^8,15^, indicating that the perception of an outcome of action as positive or negative might affect the experience of causation even after it occurred. Interestingly, the existence of a postdictive inferential strategy that prioritizes averted over direct gaze shift has been already demonstrated by Binetti and colleagues^34^, when investigating Temporal Order Judgments (TOJs) of gaze shifts behaviors to evaluate the impact of gaze direction (i.e., direct vs. averted gaze) on TOJs performance measures. Their findings showed that averted gaze shifts, as compared to mutual, determine prior entry effects, resulting in participants’ biased TOJs in favor of the eye performing the averted shift. Interestingly, the authors proposed that averted gaze shifts are prioritized because they could potentially signal the presence of behaviorally relevant information, which individuals should focus their attention on, in the environment^34^. It is plausible that this prioritization of averted over mutual gaze affects also SoA in a retrospective way, thus enhancing individuals’ feeling of control over a social signal that might signal some relevant changes in the environment.

There are two last aspects to consider. First, the lack of significant effect of the type of emotion (happy vs. sad face) in Experiment 1 remained also in Experiment 3. These results suggest that the robot’s facial expression, conveyed by its facial expression, does not affect the postdictive component in a similar way as the gaze type does, since, in Experiment 3, we did not find any significant effect of emotional expression. It might be that emotional expression does not affect SoA or that the robot we used was not sufficiently expressive to elicit the effects of interest or that specific parameters of our design attenuated the effects of emotional valence of action outcomes on sense of agency that have been reported in previous literature^8,9,15^. This latter possibility seems to be quite plausible. One explanation might be that, in those earlier studies, participants were required to estimate the onset of the emotional event, whereas in Experiment 3 participants estimated the temporal interval between the onset of their movement and the offset of the emotional event. Indeed, the “Go” utterance of the robot (the critical event to judge) occurred at the end of the trial sequence, i.e., after the robot already displayed one of the emotional facial expression (happy vs. sad face). However, it is important to point out that this explanation cannot account for all the present findings, as in Experiment 1, participants judged the onset of the emotional event, i.e., when the robot displayed the happy versus the sad face, and results still showed no significant differences between the two on participants’ temporal estimates. Thus, perhaps also the robot’s limited emotional expressiveness was responsible for the lack of the effect of interest.

Another difference between our design and the previously reported studies is that the present study employed the temporal interval estimation paradigm^5,31^, whereas the previous studies used the Intentional Binding (IB) paradigm based on the Libet clock method^46^. Although both methods were designed to measure implicit SoA, the Libet clock method seems to be more robust and sensitive to capture implicit SoA, relative to the interval estimation method (see^64^ for more information related to the differences between the methods). Therefore, it might be plausible that, in our study, the temporal estimation paradigm was not able to capture the effect of the emotional expressions on participants’ SoA, which would potentially have been the case if we employed the IB paradigm based on the Libet clock method. However, this explanation would need to be tested in future research (and, potentially, using also other robot types).

Finally, results of Experiment 3 suggested that there is no combined effect of the type of emotion (happy vs. sad face) and type of gaze (mutual vs. averted gaze) on participants’ implicit SoA, but only a main effect of averted gaze as compared to the mutual gaze. This, in turn, would suggest that the robot’s gaze is a more potent factor than facial expression in modulating participants’ SoA.

Taken together, our findings suggest that emotional facial expressions, in the form of robot’s happy versus sad face do not affect SoA. Conversely, communicative gaze (i.e., both mutual and averted gaze) modulated SoA by means of affecting the postdictive mechanisms underlying SoA.

## Supplementary Information

Supplementary Information.

## Data Availability

Datasets used for the analyses, Supplementary Materials file, and videos of all the three experiments can be found at the following link: https://osf.io/ktd58/?view_only=1b35187d122145dc89c7a47f8f62ac12 (OSF repository name: “The impact of facial expressions and eye contact of a humanoid robot on individual Sense of Agency”).
