# Supplementary material for: The impact of facial expression and communicative gaze of a humanoid robot on individual Sense of Agency
Source: Sci Rep. 2023 Jun 21;13:10113. doi: 10.1038/s41598-023-36864-0 (PMC10284854; doi:10.1038/s41598-023-36864-0)
Supplement: Supplementary file 1 — Supplementary Information. [file 41598_2023_36864_MOESM1_ESM.docx]

Supplementary Materials for

**The impact of facial expression and communicative gaze of a humanoid robot on individual Sense of Agency**

Maria Lombardi^1 †^, Cecilia Roselli^1†^, Kyveli Kompatsiari^1^, Federico Rospo^1^, Lorenzo Natale^1^, Agnieszka Wykowska^1*^

^1^ Italian Institute of Technology, Via Morego 30, 16163, Genoa, Italy

^*^ Corresponding author: Agnieszka Wykowska

Italian Institute of Technology

Via Morego 30

16163 Genova, Italy

Phone number: + 39 0108172242

E-mail: [Agnieszka.Wykowska@iit.it](file:///D:\Instance_EEG_Experts\DraftsForPaper\Agnieszka.Wykowska@iit.it)

**This file includes:**

**SM.1. *Z*-transformed scores: rationale**

**SM.1.1. Experiment 1**

**Figure SM.1.1.** Participants’ mean temporal estimates, in form of z-transformed scores, plotted as a function of outcome type (happy vs. sad face). Black dots represent the mean value for each outcome type, whereas colored dots represent individual means.

**SM.1.2. Experiment 2**

**Figure SM.1.2.** Participants’ mean temporal estimates, in form of *z-*transformed scores, plotted as a function of outcome type (mutual vs. averted gaze). Black dots represent the mean value for each outcome type, whereas colored dots represent individual means.

**SM.1.3. Experiment 3**

**Figure SM.1.3.** Participants’ mean temporal estimates, in form of *z-*transformed scores, plotted as a function of Gaze (mutual vs. averted gaze). Black dots represent the mean value for each outcome type, whereas colored dots represent individual means.

**SM.1.4. Conclusions**

**References**

**SM.1. *Z*-transformed scores: rationale**

As participants may differ in the way they used the Likert-like scale to provide their response in the time interval estimation task, in all experiments we transformed participants’ raw estimates into *z-*scores. Indeed, they can reduce irrelevant inter-subject variability by subtracting from each interval estimate the mean estimate for that participant across all trials, and by dividing the resulting differences by the standard deviations of all estimates for that participant (see [1] for a similar procedure applied to the time interval estimation paradigm).

It is important to point out that, as with participants’ raw estimates, a lower *z-*score suggests a higher Sense of Agency (SoA), whereas a higher *z-*score suggests a lower SoA.

**SM.1.1. Experiment 1**

We compared the mean values of participants’ temporal estimates, now transformed into *z-*scores.

Since the data did not meet the assumption of normality, which was assessed through the Shapiro-Wilk test (p = 0.001), we performed a non-parametric Wilcoxon signed-rank test in JASP 0.14.1.0 (2020). The threshold of significance level was set at p < 0.05; rank-biserial coefficient (r_b_) is reported as an index of the effect size; 95% confidence intervals are reported.

Results did not show any significant difference, in terms of participants’ SoA (i.e., temporal estimates), between the two types of outcome (happy vs. sad face) [W = 121, p = 0.42, r_b_ = -0.19, 95% CI = (-0.57; 0.26); Mean _Happy_ = 0.05, Mean _Sad_ = -0.04] (see **Figure SM.1.1**).


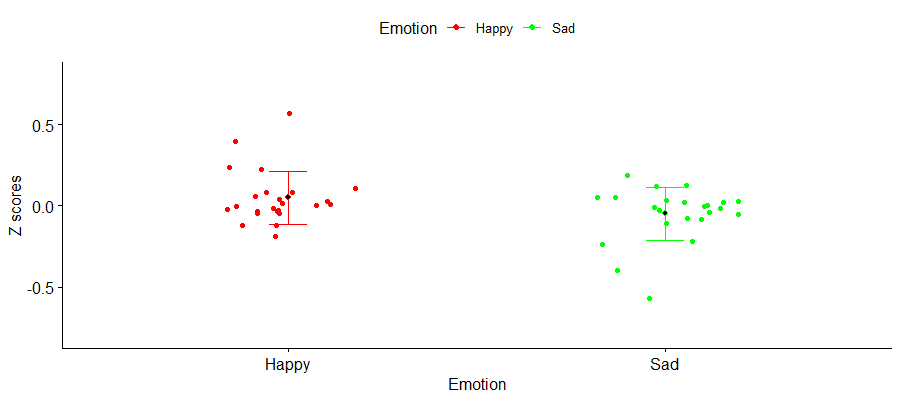


**Figure SM.1.1.** Experiment 1. Participants’ mean temporal estimates, in form of z-transformed scores, plotted as a function of outcome type (happy vs. sad face). Black dots represent the mean value for each outcome type, whereas colored dots represent individual means.

**SM.1.2. Experiment 2**

We compared the mean values of participants’ temporal estimates, now transformed into *z-*scores.

Since the data met the assumption of normality, which was assessed through the Shapiro-Wilk test (p = 0.6), we performed a paired sample t-test in JASP 0.14.1.0 (2020). The threshold of significance level was set at p < 0.05; Cohen’s d is reported as an index of the effect size; 95% confidence intervals are reported.

Results showed a significant difference, in terms of participants’ temporal estimates, as a function of the type of outcome (mutual vs. averted gaze) [t = 11.97, p < 0.001, Cohen’s d= 2.3, 95% CI = (1.56; 3.02); Mean _Mutual_ = -0.5, Mean _Averted_ = 0.4] (see **Figure SM.1.2**).

**
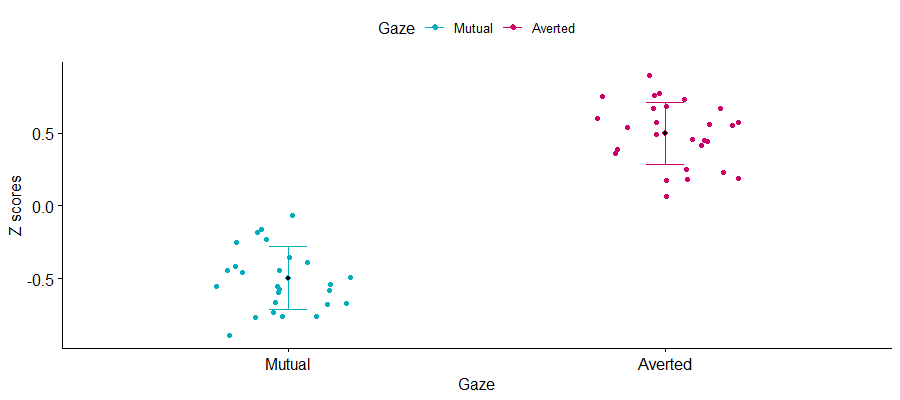
**

**Figure SM.1.2.** Experiment 2. Participants’ mean temporal estimates, in form of *z-*transformed scores, plotted as a function of outcome type (mutual vs. averted gaze). Black dots represent the mean value for each outcome type, whereas colored dots represent individual means.

**SM.1.3. Experiment 3**

As in the previous experiments, we used *z-*transformed scores to assess whether participants were not biased by their use of the rating scale to provide their answers regarding the temporal estimates. Thus, as in the main manuscript, we performed two separate 2 (Emotion: happy, sad) X 2 (Gaze: mutual, averted) ANOVAs in JASP 0.14.1.0 (2020), according to the type of factor (emotion/gaze) which was blocked or randomized. Participants’ temporal estimates, in the form of *z*-scores, were the dependent variable, and the type of emotion and gaze were the within-subjects factors. The threshold for level of significance was set at p < 0.05; η^2^ is reported as an index of the effect size.

*Emotion as Blocked Factor and Gaze as Randomized Factor.* Results showed that the main effect of gaze was significant [F _(1, 34)_ = 12.8, p = 0.001, η² = 0.03], with lower temporal estimates (i.e., higher implicit SoA) when the outcome of participants’ actions was the averted, relative to the mutual gaze (M _Averted_ = -0.07 ms, SE _Averted_ = -0.03; M _Mutual_ = 0.06 ms, SE _Mutual_ = 0.1) (see **Figure SM.1.3**). No other main effects or interactions resulted to be significant (all ps > 0.3).

*
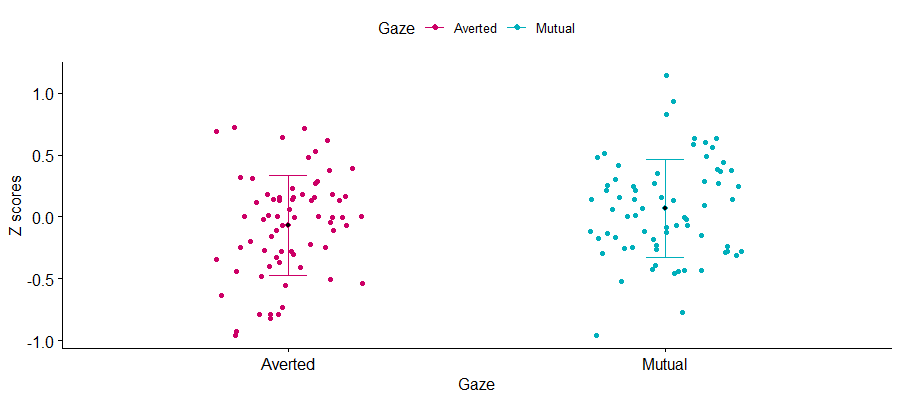
*

**Figure SM.1.3.** Experiment 3. Participants’ mean temporal estimates, in form of *z-*transformed scores, plotted as a function of Gaze (mutual vs. averted gaze). Black dots represent the mean value for each outcome type, whereas colored dots represent individual means.

*Gaze as Blocked Factor and Emotion as Randomized Factor.* Results did not show any significant main effect or interaction (all ps. > 0.07).

**SM.1.4. Conclusions**

When transforming participants’ raw temporal estimates into *z-*scores, in all the three experiments results mirrored the ones reported in the main manuscript. Specifically, in Experiment 1 no significant differences emerged in terms of temporal estimates as a function of the type of robot’s facial expression (happy vs. sad face). In Experiment 2, results showed that participants experienced a stronger implicit SoA (i.e., lower *z­*- scores of temporal estimates) when the outcome of their action was the robot’s mutual gaze, as compared to the averted gaze. In Experiment 3, results went in the opposite direction, namely, participants experienced a stronger SoA, in the form of lower temporal estimates, when the outcome of their action was the robot’s averted, as compared to mutual gaze. Notably, it occurred only when the gaze factor was randomized, and not when it was a blocked factor.

**References**

[1] Caspar, E. A., Lo Bue, S., Magalhães De Saldanha da Gama, P. A., Haggard, P., & Cleeremans, A. The effect of military training on the sense of agency and outcome processing. Nat. Commun., 11(1), 1-10 (2020). <https://doi.org/10.1038/s41467-020-18152-x>
